# Donor, Acceptor, and Molecular Charge Transfer Emission All in One Molecule

**DOI:** 10.1021/acs.jpclett.2c03925

**Published:** 2023-03-10

**Authors:** Larissa G. Franca, Andrew Danos, Andrew Monkman

**Affiliations:** Department of Physics, Durham University, South Road, Durham DH1 3LE, United Kingdom

## Abstract

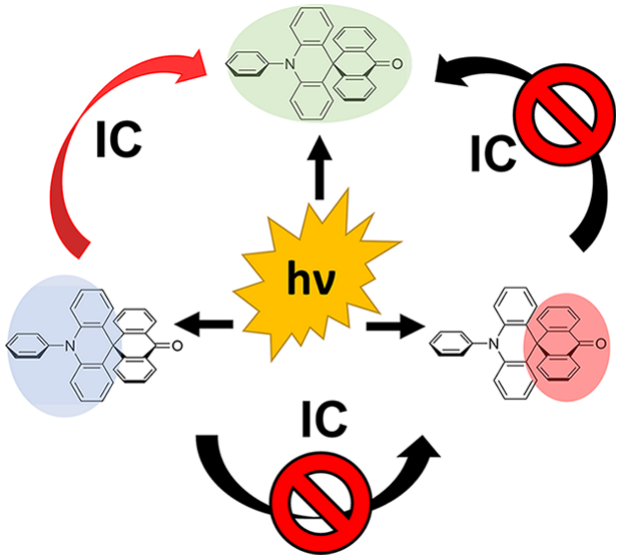

The molecular photophysics in the thermally activated delayed fluorescence (TADF) spiro-acridine–anthracenone compound, ACRSA, is dominated by the rigid orthogonal spirocarbon bridging bond between the donor and acceptor. This critically decouples the donor and acceptor units, yielding photophysics, which includes (dual) phosphorescence and the molecular charge transfer (CT) states giving rise to TADF, that are dependent upon the excitation wavelength. The molecular singlet CT state can be directly excited, and we propose that supposed “spiro-conjugation” between acridine and anthracenone is more accurately an example of intramolecular through-space charge transfer. In addition, we show that the lowest local and CT triplet states are highly dependent upon spontaneous polarization of the environment, leading to energy reorganization of the triplet states, with the CT triplet becoming lowest in energy, profoundly affecting phosphorescence and TADF, as evident by a (thermally controlled) competition between reverse intersystem crossing and reverse internal conversion, i.e., dual delayed fluorescence (DF) mechanisms.

As an efficient method to harvest typically non-emissive triplet excitons in organic light-emitting diodes (OLEDs), thermally activated delayed fluorescence (TADF)^1^ has attracted great interest as a means to do so without the need for rare and expensive heavy metals.^2,3^ To achieve efficient thermal activation of reverse intersystem crossing (rISC), TADF molecules must meet several key criteria; foremost among these is a small S_1_–T_1_ energy gap (Δ*E*_ST_), typically <50 meV, resulting from minimal electron exchange interaction energy.^4−6^ One way to achieve this is by engineering molecules with lowest energy excited states of charge transfer (CT) character, which gives effective separation of electron and hole wave functions and, therefore, vanishing exchange energy.^4,7,8^ This outcome is achievable through either linking structurally orthogonal electron donor (D) and acceptor (A) units (sterically maintaining a decoupling of their individual electronic systems) or a large spatial separation of D and A molecules as in through-space charge transfer molecules or simple bimolecular mixtures (exciplexes).^9^

However, when the electron exchange energy approaches zero, the singlet (^1^CT) and triplet (^3^CT) orbitals become fully degenerate and, thus, transitions between the two become forbidden because no necessary change in orbital angular momentum can occur.^10^ To facilitate rISC (and ISC),^10^ a second triplet excited state, very close in energy to the ^3^CT state but having a different orbital character, is required. Through non-adiabatic mixing via vibronic coupling with ^3^CT, this second triplet excited state mediates the required spin flip and couples the CT triplet back to the singlet manifold. This coupling state can be either a local triplet state (^3^LE) or a higher lying triplet CT state.^11^

An alternative method to achieve the same outcome is reverse internal conversion (rIC). This can become an efficient triplet upconversion mechanism when an upper triplet state (T_N_) is isoenergetic to S_1_ and the energy gap between T_1_ and T_N_ can be crossed by thermal excitation.^12^ This mechanism does not require vibrational mixing of triplet states to enable the rISC step and is thought to be responsible for TADF in multi-resonance B,N-doped nanographenes.^13−15^

To achieve such highly decoupled donor and acceptor electronic systems in a molecule, an orthogonal donor–acceptor bridging bond is used. Typically a N–C bond gives the appropriate energy level ordering, perpendicular structure, and very small exchange energy, although other linking strategies are successfully though less frequently employed.^2,3,16−18^ Spiro-linked D–A molecules, bridged rigidly and orthogonally about a spiro-center, can also yield efficient rISC.^19−21^ Other ways to achieve decoupled D and A include the use of an inert scaffold to separate D and A units, exemplified by the triptycene-bridged acridine–triazine donor–acceptor TADF molecule TpAT–tFFO reported by Wada et al.^22^ The spiro-linkage is distinct from single-bond C–N linkages, which can rotate and allow the linked donor and acceptor fragments to take up a distribution of angles, leading to a wide range of rISC rates, i.e., highly disordered TADF systems.^23^ Thus, alongside their TADF performance, spiro TADF systems offer reduced inhomogeneous complexity in their delayed emission kinetics, which is valuable for studying the intrinsic photophysics of TADF.^24^

As an exemplar system, the spiro-linked acridine–anthracenone derivative 10-phenyl-10*H*,10′*H*-spiro[acridine-9,9′-anthracen]-10′-one (ACRSA; Figure S1 of the Supporting Information) was one of the first reported spiro-linked TADF materials. It has high photoluminescence efficiency of 85%,^25^ with good OLED performance doped in the host bis[2-(diphenylphosphino)phenyl] ether oxide (DPEPO), achieving an external quantum efficiency (EQE) of 16.5%.^25,26^ We have recently studied the photophysics of this material in solution^27^ and solid state^24^ to understand the rISC mechanism and examine the homogeneity of rISC rates controlled by the rigid spiro D–A bridging bonds. In these previous works, we uncovered the complex nature of the photophysics and, surprisingly, multiple electronic energy levels involved in ISC and rISC of this material, which totally disobeys Kasha’s rule.

As a consequence of the critically tuned electronic decoupling of ACRSA imposed by the rigid spiro D–A bridging bonds, here, we demonstrate that we can elicit photophysical behavior in the molecule corresponding to either the donor or acceptor subunit as well as the full molecular CT state, dependent upon the excitation wavelength. This is particularly highlighted by the discovery of excitation-dependent phosphorescence. We hence draw the conclusion that, in the spiro D–A system, charge transfer states are more intramolecular through space in nature than through bond.

Finally and also as a consequence of the critically minimized D–A coupling, we experimentally demonstrate competing dual delayed fluorescence (DF) mechanisms in ACRSA. This comes about through spontaneous reordering of the highly environment-sensitive lowest triplet states. In nonpolar environments a local triplet state is lowest in energy, giving rise to phosphorescence, typical vibronically coupled TADF, and at higher temperatures, rIC upper state crossing DF. All three mechanism are in competition, and we demonstrate a critical temperature at which rIC-driven rISC becomes more efficient than vibronic coupling rISC. In moderately polar solvents or high polarizability hosts the energy ordering flips, and the lowest energy triplet state is instead a CT triplet state. In this case, ^3^CT phosphorescence decay is highly forbidden and suppressed, and therefore, vibronic coupling rISC is free to harvest most triplet states with only moderate temperature dependence. Because there is little or no population in the (higher energy) lowest local triplet state, rIC also becomes ineffective. These observations show for the first time how sensitive TADF is to this triplet energy ordering.

ACRSA has two main absorption bands in the ultraviolet (UV) (ε > 10^5^ M^–1^ cm^–1^), which theoretical calculations^28^ identify as a pair of ππ* transitions: 1^1^B_1_ at 340 nm (3.64 eV) and 2^1^B_2_ at 324 nm (3.83 eV) (see Figure S3 of the Supporting Information). In comparison to the experimental absorbance or excitation spectra of acridine D and anthracenone A units (Figure 1c), the acridine unit absorbs at 337 nm (3.7 eV) and emits characteristic UV emission, identifying the 1^1^B_1_ state in ACRSA as corresponding to the D ππ* transition. The acceptor anthracenone (or anthrone) unit is found to absorb at around 300–400 nm (Figure 1d) and emits a well-structured luminescence at wavelengths above 400 nm, very similar to that seen from ACRSA when excited at 355 nm. This identifies the nπ* state of A, ascribed as a 2^1^A_1_ transition (Figure S3 of the Supporting Information).^27^ We therefore also conclude that the 2^1^B_2_ state is the anthracenone ππ* state. However, when ACRSA is excited at either 330 or 350 nm, we also observe a strong Gaussian-shaped emission at 500 nm, indicative of a CT state. The full excitation-dependent emission is shown in the contour plot (Figure 1e). The CT nature of the Gaussian emission is confirmed by its strong solvatochromism (Figure S4 of the Supporting Information), and its intensity increases by an order of magnitude upon removal of dissolved oxygen, indicating the highly efficient rISC of triplets through this CT state.^27^ Indeed, it is only with the aid of oxygen quenching of the CT band DF that we are able to resolve the separate and much weaker donor or acceptor emission bands (Figure S5 of the Supporting Information). The donor and acceptor emission bands lie beneath the ^1^CT contribution. In the degassed environment, ^1^CT totally dominates emission through the efficient rISC harvesting of the triplet states. Therefore, with the addition of oxygen, we quenched the DF (rISC) mechanism, consequently identifying the weak emission signals coming from both donor and acceptor units, otherwise hidden by the very strong DF CT emission.

**Figure 1:**
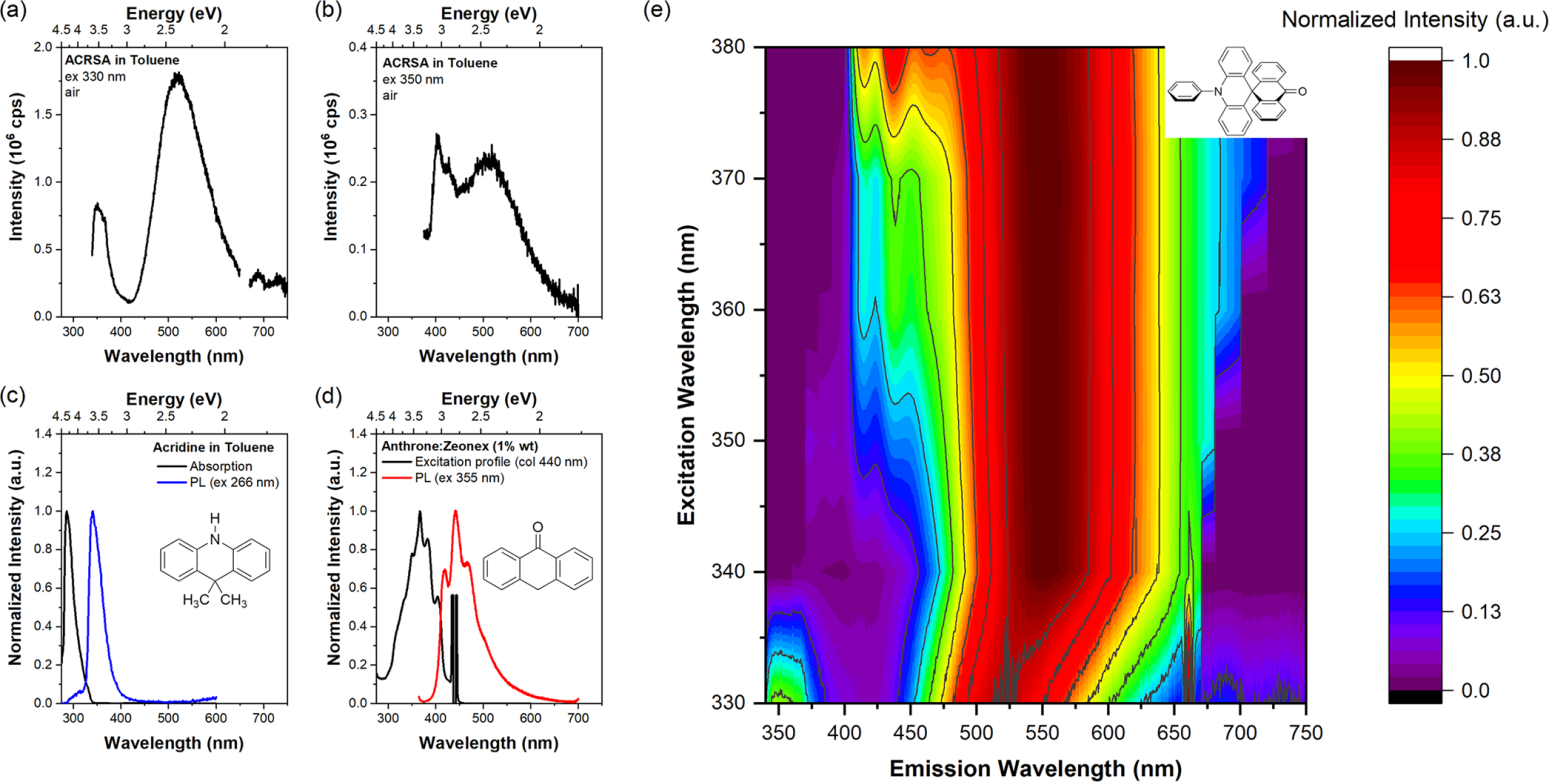
Photoluminescence (PL) spectra of ACRSA in toluene (air-equilibrated, 50 μM), with excitation at (a) 330 nm and (b) 350 nm. (c) Absorption and PL spectra of the acridine unit (donor unit) in toluene. (d) Excitation and PL spectra of the anthracenone unit (acceptor unit) in the zeonex matrix at 1 wt % concentration, with excitation collected at 440 nm marked with a black line. (e) Contour plot of PL spectra of ACRSA in toluene (air-equilibrated, 50 μM), exciting at different wavelengths. The contour plot was interpolated from Figure S2 of the Supporting Information.

To identify any direct CT absorption and emission, excitation profiles were measured using different emission collection wavelengths and are shown in the excitation contour plot (Figure 2). Exciting into the acridine ππ* band (from 300 to 340 nm) leads to very strong CT formation and emission at ∼500 nm. Excitation at energies below this state (wavelengths above 340 nm) also directly photoexcites CT states but yields much lower intensity emission. This indicates the presence of a direct CT absorption, between 350 and 400 nm, but which is much weaker than the anthracenone nπ* transition. From these wavelength-dependent measurements, it is clear that when exciting the acridine ππ* state, the excitation energy cannot access the orthogonally oriented anthracenone nπ* state (Figure 1a). As a result of this effect, no contribution of anthracenone nπ* state emission is observed, indicating that IC between these two states is effectively forbidden by the orthogonality of the D and A units. Moreover, when the anthracenone nπ* transition is excited, this state does not internally convert to the ^1^CT state. As previously shown by femtosecond photoinduced absorption measurements, the anthracenone nπ* state instead rapidly undergoes El-Sayed-allowed ISC to the anthracenone triplet (1^3^A_1_) ππ* state.^27^ We believe that the direct CT absorption overlaps with the anthracenone nπ* transition (in nonpolar environments) such that emission from both states appears simultaneously, despite no transfer of excitation between these states (Figure 1b). A diagram representing these transitions is shown in Figure S6 of the Supporting Information.

**Figure 2:**
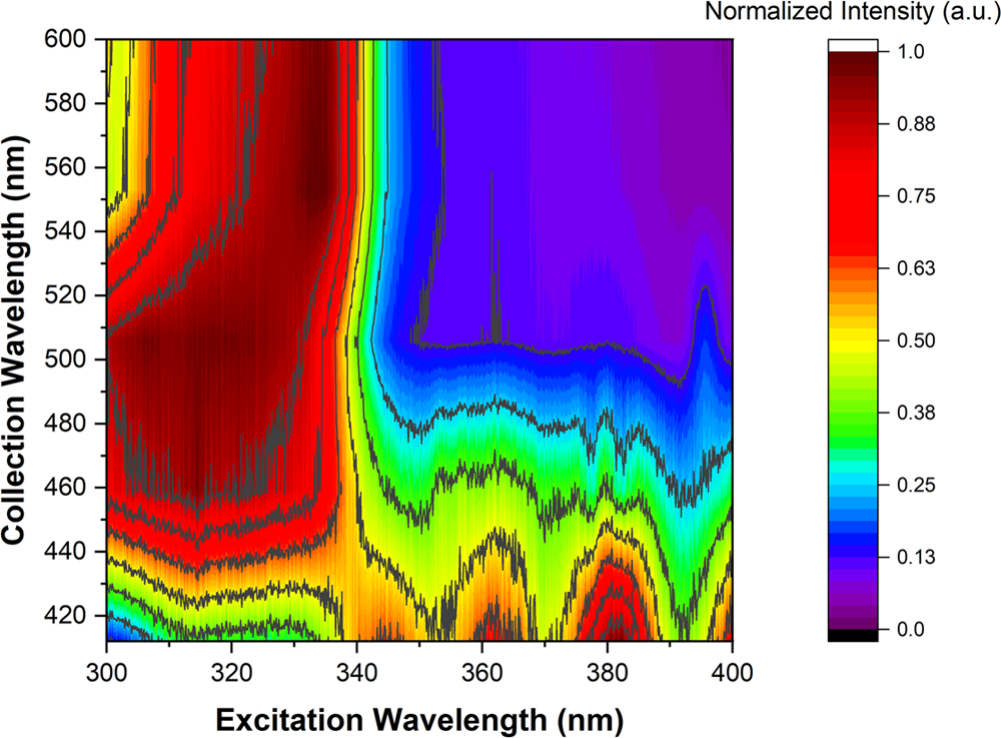
Contour plot of excitation spectra collected at different wavelengths of ACRSA in toluene (air-equilibrated solution with a concentration of 50 μM). The contour plot was interpolated from Figure S7 of the Supporting Information.

From time-resolved measurements in degassed toluene solutions (Figure S8 of the Supporting Information), it is clear that both 337 and 355 nm excitation lead to very strong DF signals, indicating that triplet production from both the acridine (1^1^B_1_) ππ* and anthracenone (2^1^A_2_) nπ* singlet states is highly efficient (Figure S6 of the Supporting Information).

To identify the lowest lying triplet state, low-temperature phosphorescence measurements were made on films of ACRSA dispersed in zeonex at 1% (w/w) concentration. Very surprisingly, the measured strong phosphorescence is also excitation-dependent (Figure 3). With excitation at 355 nm into the anthracenone (2^1^A_2_) nπ* singlet state (Figure 3a), we identified well-structured phosphorescence observed after 22 μs at 80 K, as we previously reported,^27^ characteristic of the anthracenone (1^3^A_2_) nπ* triplet state. The vibronic progression spacing of 190 meV in this phosphorescence spectra is identified as arising from strong C=O vibrational coupling to the electronic state (Figure 3).^27^ We also observe a high energy knee (at 420 nm) that stands apart from the 190 meV vibronic progression, which we ascribe to emission from the higher energy anthracenone (2^1^A_2_) ππ* triplet state in weak thermal equilibrium with the (1^3^A_2_) nπ* triplet state.

**Figure 3:**
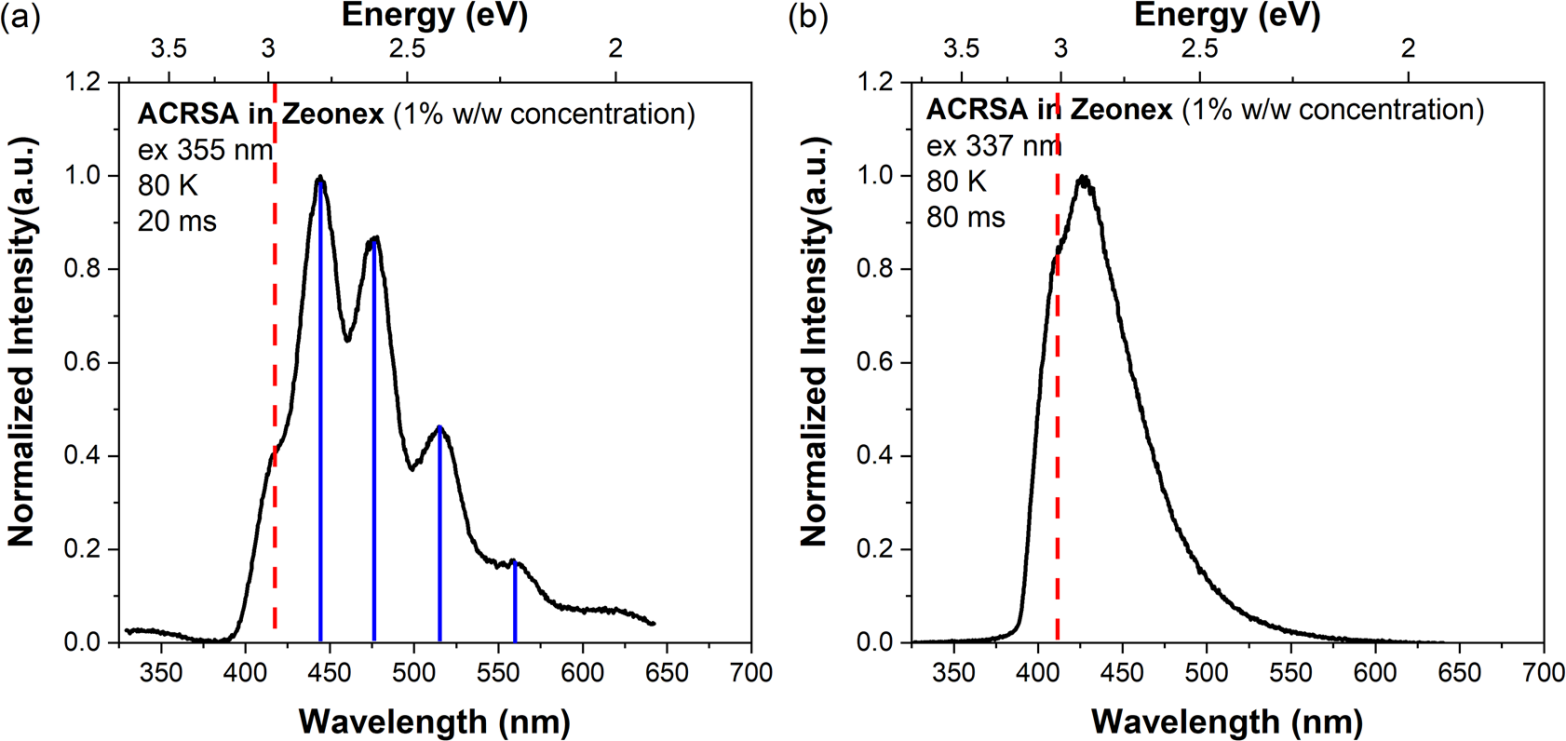
Phosphorescence spectra obtained at 80 K of ACRSA in zeonex at 1% concentration, excited at (a) 355 nm and (b) 337 nm.

Excited at 337 nm into the acridine ππ* band, however, gives a completely different phosphorescence spectrum, again having high intensity (Figure 3b). Now, the phosphorescence is less vibrationally structured, and the emitting state is of higher energy than the anthracenone (2^1^A_2_) ππ* triplet state. This we ascribe to emission from the acridine (1^3^B_2_) ππ* triplet state. Given that this phosphorescence persists for 80 ms without being eventually quenched by the shorter lived, highly structured, lower energy anthracenone (1^3^A_2_) nπ* triplet state, it is clear that the acridine and anthracenone subunits are highly electronically decoupled. An energy diagram representing the pathways for the decay of the triplet excited state is shown in Figure S9 of the Supporting Information.

Moreover, the strong phosphorescence of both the donor and acceptor triplet states suggests that they do not couple to the molecular CT states at a low temperature. This could arise from negligible orbital overlap between states and very weak spin–orbit coupling (SOC) between them as well.^28^ The various excitation-dependent energy decay pathways in ACRSA are summarized in the energy level schemes shown in Figure S10 of the Supporting Information.

When the solvent polarity is changed, a rearrangement in the energy levels is observed, as expected. From the changes of the relative energy positions of the singlet and triplet states, we can understand the lifetime decays of the various singlet states, especially the ^1^CT state, which shows rather anomalous behavior. Independent of the excitation wavelength (and so purely an effect of the ^1^CT lifetime), the prompt ^1^CT lifetime is found to be 8 ns in methylcyclohexane (MCH), 274 ns in toluene, and 75 ns in dichloromethane (DCM) (Figure S12 of the Supporting Information). In MCH, ^1^CT ππ* is energetically above the ^3^LE_A_ nπ* triplet state, and thus, El-Sayed-allowed ISC competes with radiative decay, which effectively quenches the ^1^CT state to the triplet manifold. In toluene, the ^1^CT state is relaxed by the solvent polarity, and instead, this state is energetically below ^3^LE_A_ nπ*. Thus, the ISC channel is greatly reduced, and therefore, the radiative decay of ^1^CT is far less quenched with a longer corresponding lifetime. Indeed, a relatively long singlet radiative lifetime of around 200 ns is very reasonable for a ^1^CT state, highly decoupled from the ground state as in ACRSA. In DCM, ^1^CT is shifted far to the red (lower in energy) and the radiative lifetime is now reduced by non-radiative decay following the inverse energy gap law. Therefore, the radiative lifetime of the ^1^CT state maps out the relative energy separation of the ^1^CT and lowest energy ^3^LE_A_ nπ* local triplet states as a function of environmental polarity.

The mechanisms that describe the different ISC and rISC pathways with excitation dependence are summarized in Scheme S1 of the Supporting Information. The coupled C=O vibrational mode observed from the phosphorescence of the anthracenone (1^3^A_2_) nπ* triplet state is theoretically determined to be the mode responsible for vibrational coupling between the anthracenone (1^3^A_2_) nπ* triplet and ^3^CT (2^3^A_2_) states,^28^ which gives rise to high rISC rates of order of 6 × 10^5^ s^–1^ in toluene solution^27^ and exceeding 10^6^ s^–1^ in the solid state.^24^

The delayed CT emission is 10-fold more intense than the prompt CT emission (Figure S12 of the Supporting Information). Large DF contributions are also observed in many exciplex TADF molecules^29^ and especially intramolecular through space TADF systems, where the D and A are physically decoupled spatially but TADF is still driven by second-order vibrational coupled SOC and efficient.^30^

In combination with the strongly decoupled nature of the D and A localized excited (LE) states (as demonstrated above in singlet and triplet emission channels), we argue that the CT states in ACRSA are also intramolecular through space charge transfer-like rather than the expected through-bond conjugation. This realization gives deep and novel insight into the nature of “spiro-conjugation”^31^ that provides orbital overlap of D and A, in that the spiro-center does not contribute beyond tethering the D and A fragments at distances and orientations that allow for a through-space interaction.^32−34^ We have previously shown how spiro-conjugation can enhance intramolecular charge transfer though the formation of electronically isolate trap states, with this isolation enforced by the weak orbital interaction across the spiro-center.^35^ Such an intramolecular through space CT TADF has also recently been shown to be very efficient in the triptycene-bridged acridine–triazine donor–acceptor TADF molecule TpAT–tFFO.^20,33^

We further examined the excitation-dependent photophysics of ACRSA in solid films using zeonex and DPEPO hosts (Figure S13 of the Supporting Information). We do not observe significant differences in the kinetics of the time-resolved photoluminescence decays with different excitation wavelengths, although small differences in DF intensity are observed in DPEPO, while different spectral contributions are found in zeonex time-resolved spectra. ACRSA:zeonex at 1% concentration displays a substantial donor contribution when initially excited at 337 nm. This 350 nm emission from the 1^1^B_1_ excited state decays within the first few nanoseconds in the film. We also observed an instantaneous broad emission band centered at 525 nm with both excitations (337 and 355 nm). As previously discussed, we attribute this emission to the metastable CT state arising from a conformational distortion of the peripheral phenyl ring interacting with the donor.^24^ In DPEPO, the donor emission is not detectable when excited using 337 nm. Moreover, the ^1^CT emission band shows a significant red shift when compared to the zeonex matrix. This suggest that spontaneous polarization effects of the host reorganize the energy levels of ACRSA, as described in other TADF systems.^36^ Exciting ACRSA:DPEPO with either wavelength yields a similar kinetic trend, in both cases, very different from that observed in zeonex, which ultimately corroborates that a different energy ordering exists for ACRSA in different hosts.

To more deeply understand this energy ordering, we obtained the optical singlet–triplet gap, Δ*E*_ST_, for ACRSA in both zeonex and DPEPO [at 1% (w/w) concentration; Figure S13 of the Supporting Information]. At room temperature, the energy of the ^1^CT energy state stabilizes (no further red shifting observed) after ca. 36 ns with an onset of 400 nm in the ACRSA:zeonex film. The lowest triplet state energy from the phosphorescence has onset at 412 nm (3.01 eV); thus, |Δ*E*_ST_|_zeonex_ = 93 meV. In DPEPO, ^1^CT stabilizes with an onset of 419 nm after a similar time delay. In DPEPO, we do not observe such a vibronic well-structured phosphorescence spectrum, but at long delay times, a weak phosphorescence spectrum is observed, having a similar onset of 413 nm, suggesting that this emission is also coming from the same triplet state, and thus, |Δ*E*_ST_|_DPEPO_ = 41 meV. Therefore, as in solution, the energy ordering in DPEPO changes so that both ^1^CT and ^3^CT have lower energy than the lowest ^3^LE state [anthracenone (1^3^A_2_) nπ*], characterizing this as a type III TADF emitter (Figure S16 of the Supporting Information).^37^

We also measured the DF decay at 490 nm with decreasing temperature. ACRSA in both matrices shows a longer DF lifetime at lower temperatures (Figure 4), consistent with suppression of thermally activated rISC. While ACRSA:DPEPO has monoexponential DF decay, ACRSA:zeonex lifetimes required fitting with two exponentials (Table S1 of the Supporting Information). In DPEPO, the DF lifetime increases from 9.5 μs at room temperature to 88.9 μs at 80 K. Both ACRSA:zeonex lifetimes also depend upon the temperature; τ_1_ increases from 4.6 μs at room temperature to 47 μs at 100 K, whereas τ_2_ increases from 13.7 μs at room temperature to 193.4 μs at 80 K and strongly increases in relative intensity at lower temperatures. From this, we identify τ_1_ arising from the DF, whereas τ_2_ is from the phosphorescence.

**Figure 4:**
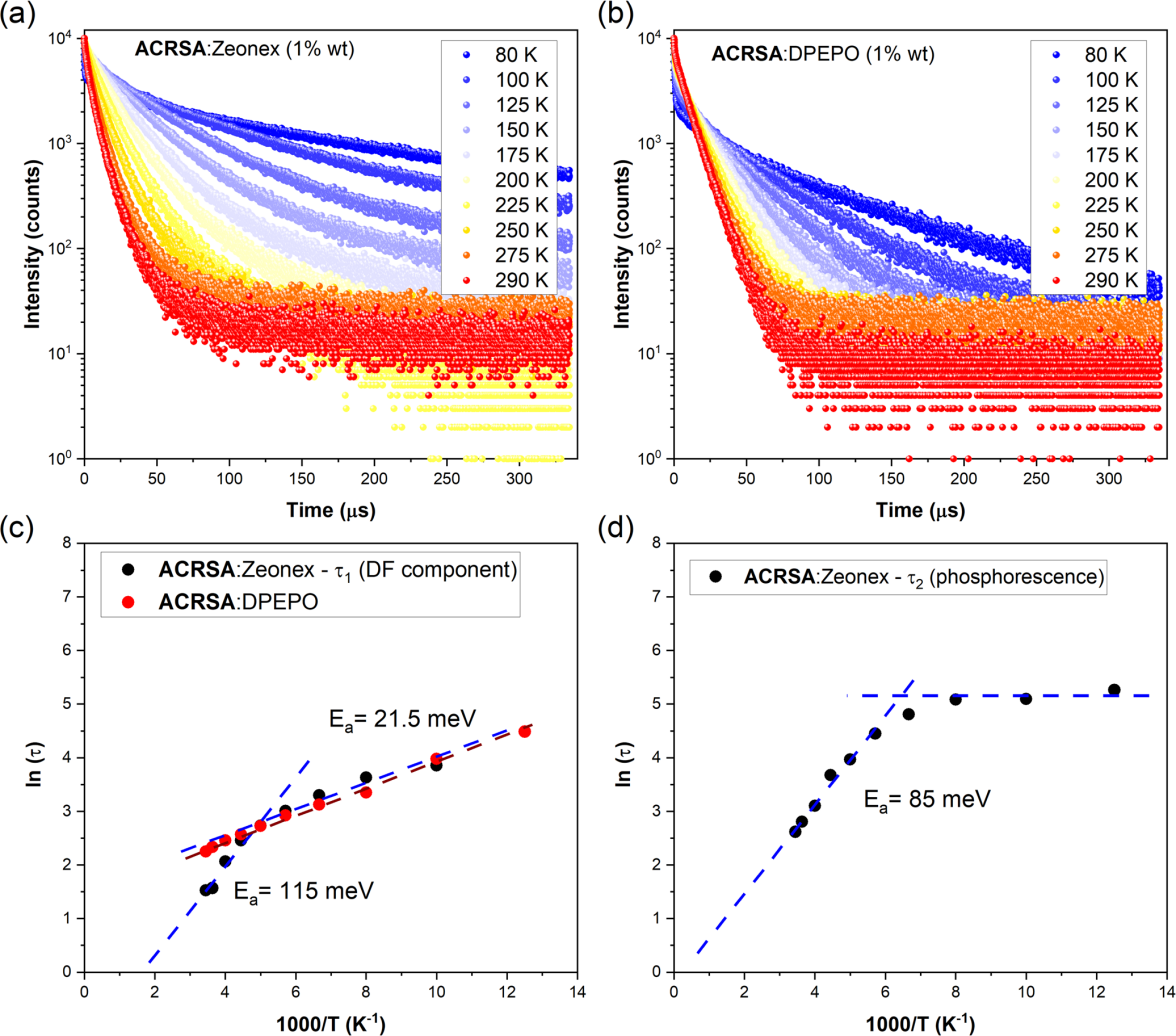
Photoluminescence decay as a function of the temperature in (a) ACRSA:zeonex and (b) ACRSA:DPEPO films (at 1% concentration), collected at 450 and 490 nm, respectively. All measurements were performed using an excitation source of 330 nm. Temperature dependence of the decay lifetime fitted using a model described elsewhere:^38,39^ (c) comparing the DF component lifetime of ACRSA:zeonex (τ_1_) and ACRSA:DPEPO and (d) phosphorescence component lifetime of ACRSA:zeonex (τ_2_).

We fitted these temperature-dependent DF lifetimes (τ_1_ component only in zeonex) using an Arrhenius model to estimate the activation energy (*E*_a_) for each process (Figure 4c). This gave an activation energy of 21.5 meV for ACRSA:DPEPO, a little lower than Δ*E*_ST_ obtained optically but in line with the predictions of Gibson and Penfold.^40^ For, ACRSA:zeonex,, we find two activation energies and a critical temperature of around 220 K. Below this critical temperature, we find a very similar behavior to ACRSA:DPEPO with an activation energy of 21.5 meV, whereas above the critical temperature, the DF becomes much faster, with the lifetime even shorter than ACRSA:DPEPO and with an activation energy of 115 meV.

From the phosphorescence spectra in zeonex, we have previously identified a small contribution from T_2_ anthracenone (1^3^A_1_) ππ*, ca. 20–50 meV higher in onset energy. Thus, the energy gap between T_1_ anthracenone (1^3^A_2_) nπ* and T_2_ anthracenone (1^3^A_1_) ππ* is estimated to be around 115–145 meV. This indicates that the high temperature activation energy represents rIC between these two anthracenone triplet states. This presence of two *E*_a_ values and regimes implies that two mechanisms give rise to DF in ACRSA:zeonex. Below the critical temperature, the DF mechanism is the same as in ACRSA:DPEPO, which, given that in DPEPO we have type III TADF behavior, confirms that this must be TADF through the second-order vibronic coupling mechanism. Indeed, Gibson and Penfold^40^ have shown that this coupling has a weak temperature dependence, as the vibronic coupling dominates. Above the critical temperature, rIC between T_1_ and T_2_ triplet states becomes effective and competitive. The population promoted into the T_2_ state can then undergo El-Sayed-allowed rISC from the anthracenone (2^1^A_2_) ππ* to singlet state, which then emits. Surprisingly, at high temperatures, the DF lifetime in ACRSA:zeonex becomes faster than that in ACRSA:DPEPO, even though Δ*E*_ST_ is bigger and the DF spectrum has the same mixed LE/CT shape as observed with optical excitation. This dual-channel DF competes more effectively with the radiative and non-radiative decays from the anthracenone (1^3^A_2_) nπ* triplet state.

In contrast, for ACRSA:DPEPO with type III TADF, the lowest triplet state is ^3^CT, which only has a very small (if any) phosphorescence rate and we presume very weak non-radiative decay to the (LE) singlet ground state. Consequently, this offers no competition to rISC, only mediated by vibronic coupling. Thus, we do not observe the rIC-mediated DF at high temperatures because all of the triplet population is in the ^3^CT state and not the anthracenone (1^3^A_2_) nπ* local T_1_ triplet state. As discussed previously, as a result of the orthogonality of the D and A units, no IC between the local anthracenone singlet and ^1^CT is observed; therefore, rIC is again not effective at populating the local T_2_ state from ^3^CT.

Lastly, plotting the τ_2_ lifetime component (phosphorescence lifetime) as a function of the temperature reveals an initially strong temperature dependence. This indicates a non-radiative quenching with an activation energy of 85 meV (685 cm^–1^) typical of high-frequency C–H modes of anthracenone. The value is also close to the rIC activation energy. Thus, we infer that there is a competition for the triplet population in T_1_ between being transferred much faster to T_2_ by rIC and decaying non-radiatively and radiatively by phosphorescence. Below ca. 150 K, this competition switches off because both rIC and non-radiative decay are suppressed and the phosphorescence lifetime approaches its natural lifetime of ca. 200 μs in this case (Figure 4d).

To conclude, the photophysical response of the spiro-acridine–anthracenone TADF molecule, ACRSA, is clearly highly excitation-wavelength-dependent. Dependent upon which initial state we photopopulate (donor, acceptor, or direct CT transition), we observe different responses and TADF channels. Further, even small changes in the environment cause reordering of energy levels, completely changing the available decay channels and efficiency of the rISC processes. This results from excitation of either the acridine donor or anthracenone acceptor subunits, which maintain a critical level of electronic decoupling, held nearly perfectly orthogonal by the rigid spiro D–A bridging bonds. We can therefore observe excitation-dependent prompt, delayed, and phosphorescence emission from either the donor or acceptor and the molecular CT state of ACRSA.

Crucially, despite the molecular CT state in ACRSA having very weak direct excitation and only coupling to the acceptor unit vibrationally, the contribution from delayed CT emission is 10-fold more intense than the prompt CT emission, which we argue implies that the CT state in ACRSA can be considered as a through-space charge transfer state rather than through bond. This insight reveals that spiro-conjugation^31^ orbital overlap of D and A is itself a through-space interaction, with the spiro-center only responsible for the spatial positioning of the D and A fragments rather than directly facilitating electronic conjugation.^32−34^

The effect of the triplet energy ordering reversal is also seen to have profound effects on the TADF. In DPEPO, the ^3^CT triplet state is lowest in energy and has highly restricted radiative and non-radiative decay routes. This state therefore acts as a metastable triplet reservoir, and we observe a simple rISC mechanism dominated by vibronic coupling and having a weak temperature dependence. In zeonex, where a ^3^LE state is lowest, we observe multiple competing DF processes. Because the lowest anthracenone (1^3^A_2_) nπ*) local triplet state in ACRSA gives efficient fast phosphorescence, at low temperatures, phosphorescence dominates and depopulates the triplet reservoir. As the temperature increases, the vibronic coupling increases the rISC rate, so that DF overtakes radiative decay from phosphorescence. At a critical temperature, ca. 220 K, we see another mechanism take over that gives even faster rISC. This we ascribe to rIC, which populates the T_2_ anthracenone (1^3^A_1_) ππ* triplet state that can readily undergo El-Sayed-allowed rISC to the anthracenone (2^1^A_2_) ππ* singlet state, which emits DF. Because it is an allowed rISC, it has a higher rate than vibronic coupling SOC, although with a larger *E*_a_, preventing it from dominating at lower temperatures. We do not observe this in DPEPO at high temperatures because all of the triplet population is in the ^3^CT state, which does not couple to the anthracenone (1^3^A_2_) nπ* local triplets as a result of larger rIC *E*_a_. Thus, the triplet energy ordering gives rise to very different TADF, and even subtle changes in the environment could cause drastic changes in the TADF mechanism.

Overall, ACRSA can and must be thought of as three molecules in one, as evidenced by the clear acceptor and donor subunits and molecular CT photophysics. This balance is achieved by critical decoupling of the D and A units associated with the spiro-center. In this picture, Kasha’s rule when applied to each subunit still holds locally but can also be disregarded when considering the photophysics of the whole molecule.

## Supporting Information Available

General experimental procedures and time-resolved photoluminescence decay (PDF)
