# Supplementary material for: Donor, Acceptor, and Molecular Charge Transfer Emission All in One Molecule
Source: J Phys Chem Lett. 2023 Mar 10;14(11):2764–71. doi: 10.1021/acs.jpclett.2c03925 (PMC10041610; doi:10.1021/acs.jpclett.2c03925)
Supplement: Supplementary file 1 — jz2c03925_si_001.pdf [file jz2c03925_si_001.pdf]

Supporting Information:

Donor, Acceptor and Molecular Charge Transfer Emission all in one Molecule

Larissa Gomes Franca, Andrew Danos and Andrew Monkman*

OEM Research group, Department of Physics, Durham University, South Road, Durham, DH1 3LE, United Kingdom.

AUTHOR INFORMATION

Corresponding Author

*Andrew P. Monkman: a.p.monkman@durham.ac.uk

Experimental section

1. **Sample preparation:**

Solutions of **ACRSA** for photophysical characterisation were prepared at low concentration of 50 µM for measurements to strictly prevent intermolecular interactions, or at a high concentration of 1 mg/mL for measurements to give an improved signal to noise. **ACRSA** was dissolved in solvents methylcyclohexane (MCH), toluene (PhMe), and dichloromethane (DCM). Degassed solutions were obtained by 5 freeze-pump-thaw cycles to remove all dissolved oxygen. Solid state samples were fabricated by drop casting onto quartz. **ACRSA** drop casted films were produced at 1% w/w in Zeonex and DPEPO (bis[2-(diphenylphosphino)phenyl]ether oxide).

2. **Photophysical characterization:**

Absorption spectra for all solutions were collected using a double beam Shimadzu UV-3600 UV/VIS/NIR spectrophotometer. Steady-state photoluminescence spectra were measured using both Jobin-Yvon Fluoromax-3 and Fluorolog spectrophotometers. Time-resolved measurements were detected by a spectrograph and a gated iCCD camera (Stanford Computer Optics), where samples were excited with a Nd:YAG laser (EKSPLA) emitting at 355 nm and a nitrogen laser emitting at 337 nm. Time-resolved decays as a function of temperature were recorded with a Horiba DeltaFlex TCSPC system using a 330 nm SpectraLED light source.

Chemical structures:

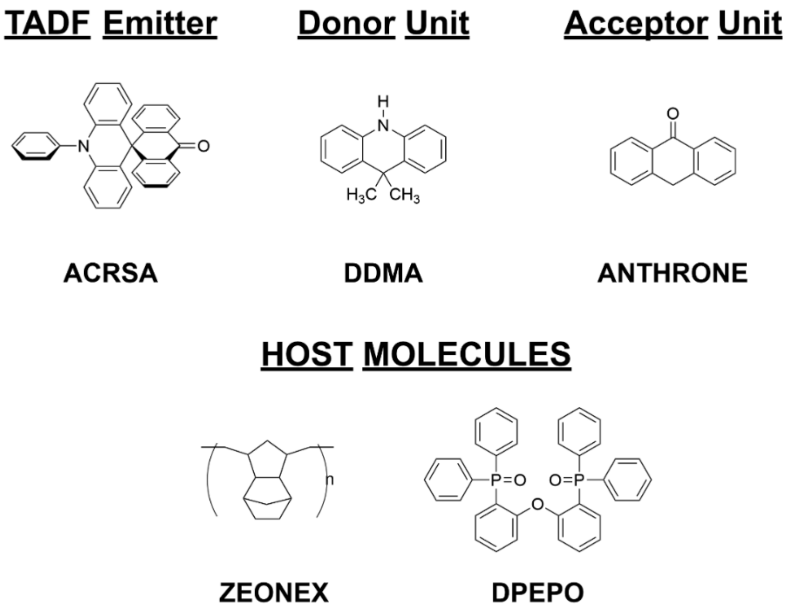

Figure S1: Chemical structures of the donor (9,10-Dihydro-9,9dimethylacridine) and acceptor (anthrone) units and host molecules used in this work.

Photophysical properties:

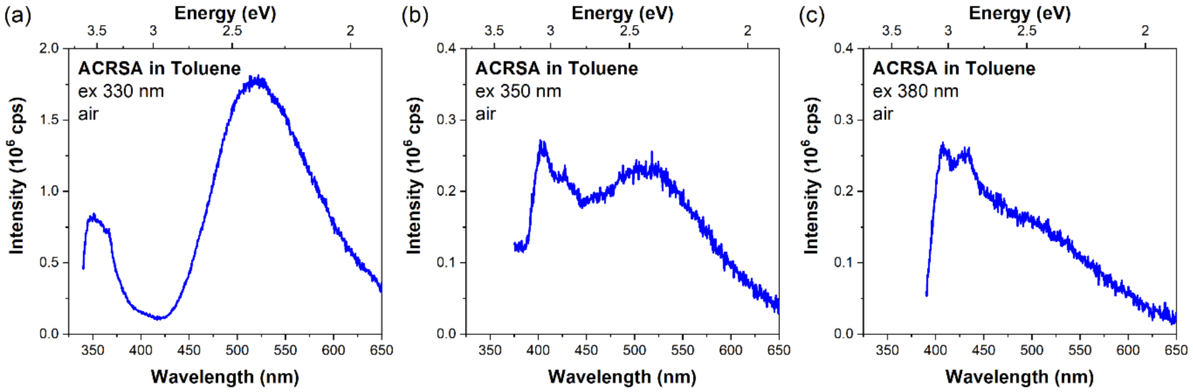

Figure S2: Photoluminescence (PL) spectra of **ACRSA** in toluene (air-equilibrated solution with a concentration of 50 µM), excitation at (a) 330 nm, (b) 350 nm and (c) 380 nm.

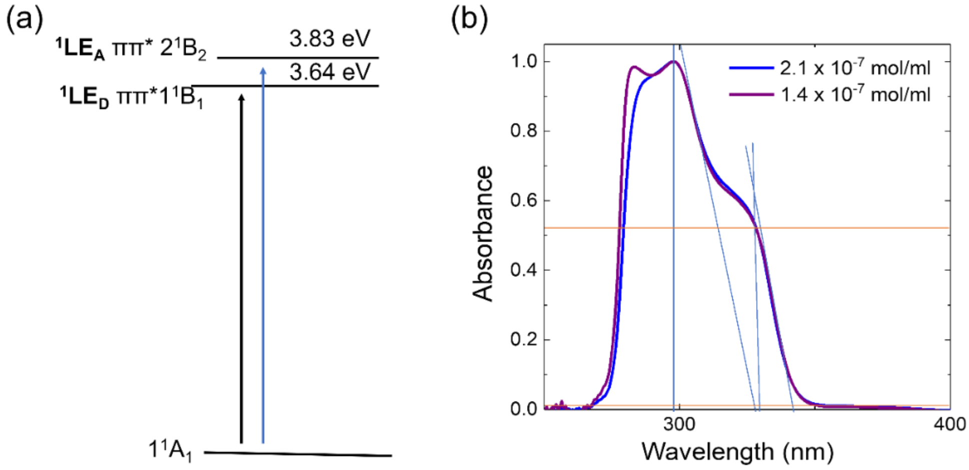

Figure S3: (a) Energy diagram representing the absorption transition of the two allowed π-π* transitions, 2^1B_2 and 1^1B_1 of **ACRSA**, from the acceptor and donor units respectively. The nomenclature used here was adopted from Lyskov and Marian.[1] (b) Normalized absorption spectra of **ACRSA** in toluene at low concentrations.

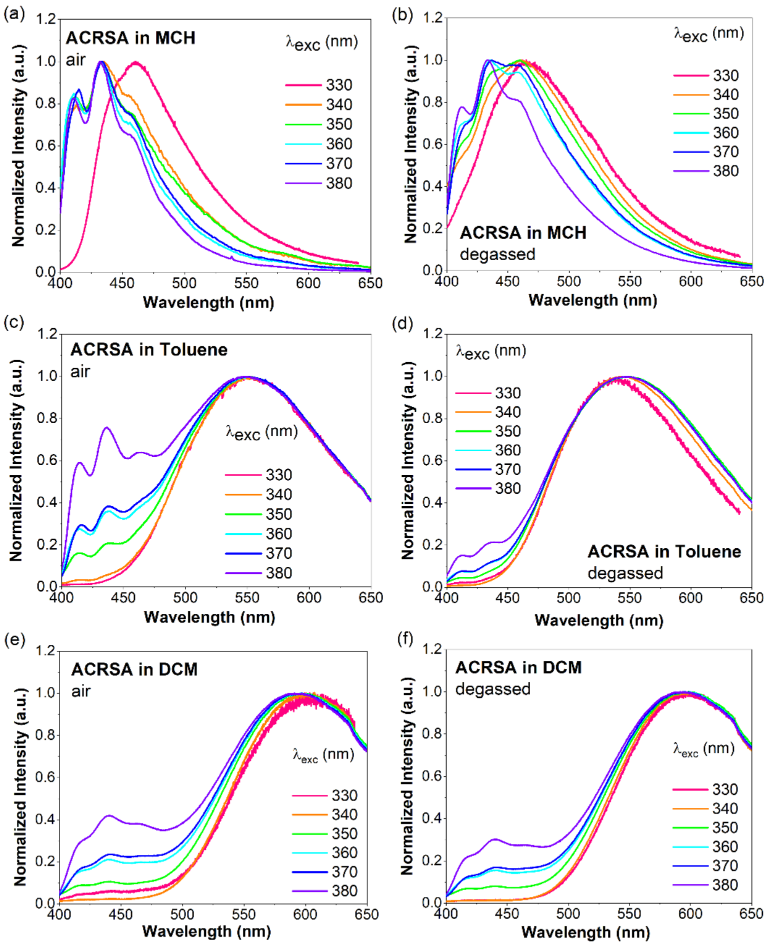

Figure S4: Normalized photoluminescence spectra of **ACRSA** (with a concentration of 1 mg/mL) in aerated and degassed solvents (a) and (b) MCH (ε = 2.02), (c) and (d) toluene (ε = 2.38), (e) and (f) DCM (ε = 8.93); at different excitation wavelengths.

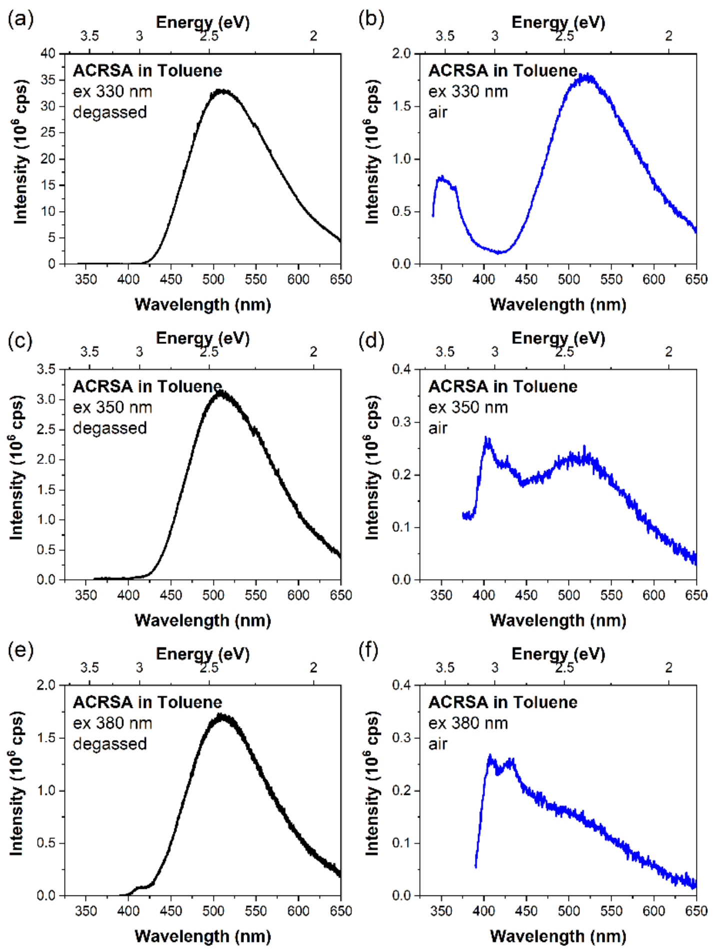

Figure S5: Photoluminescence (PL) spectra of **ACRSA** in degassed and air-equilibrated toluene solution (with a concentration of 50 µM), excitation at (a) 330 nm, (b) 350 nm and (c) 380 nm.

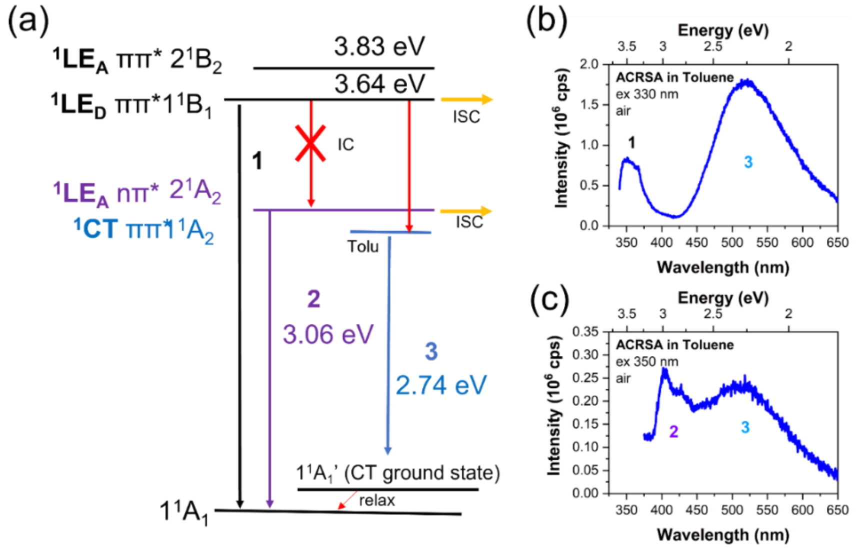

Figure S6: (a) Energy diagram representing the pathways for the decay of excited states of **ACRSA** in toluene (air-equilibrated solution with a concentration of 50 µM) when excited at different wavelengths. The nomenclature used here was adopted from Lyskov and Marian.[1] (b) Photoluminescence spectra highlighting the bands related to the represented transition.

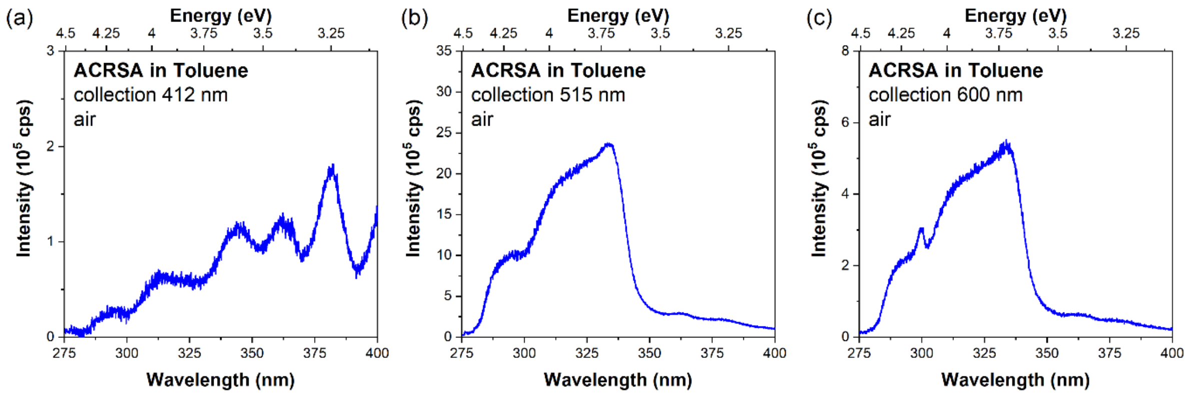

Figure S7: Excitation spectra of **ACRSA** in toluene (air-equilibrated solution with a concentration of 50 µM), collection at (a) 412 nm, (b) 515 nm and (c) 600 nm.

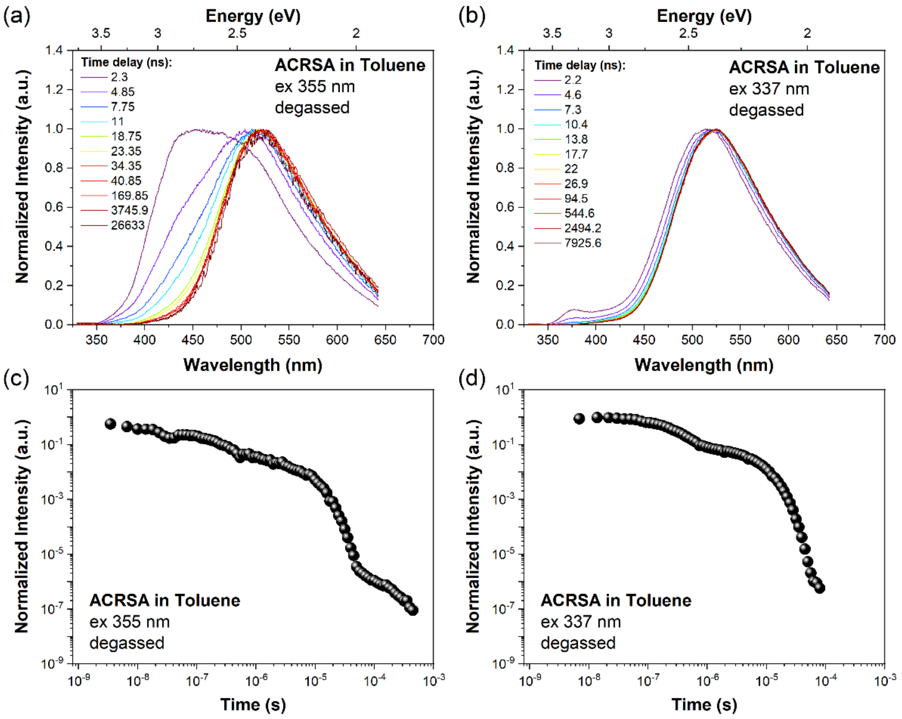

Figure S8: Normalized time resolved photoluminescence spectra and kinetic decay of **ACRSA** in toluene, excited at: (a-c) 355 nm (the direct mixed 2^1A_2 and 1^1A_2 transitions) and (b-d) 337 nm (into the 1^1B_1 transition). All measurements were performed in degassed solutions (with a concentration of 50 µM) at room temperature.

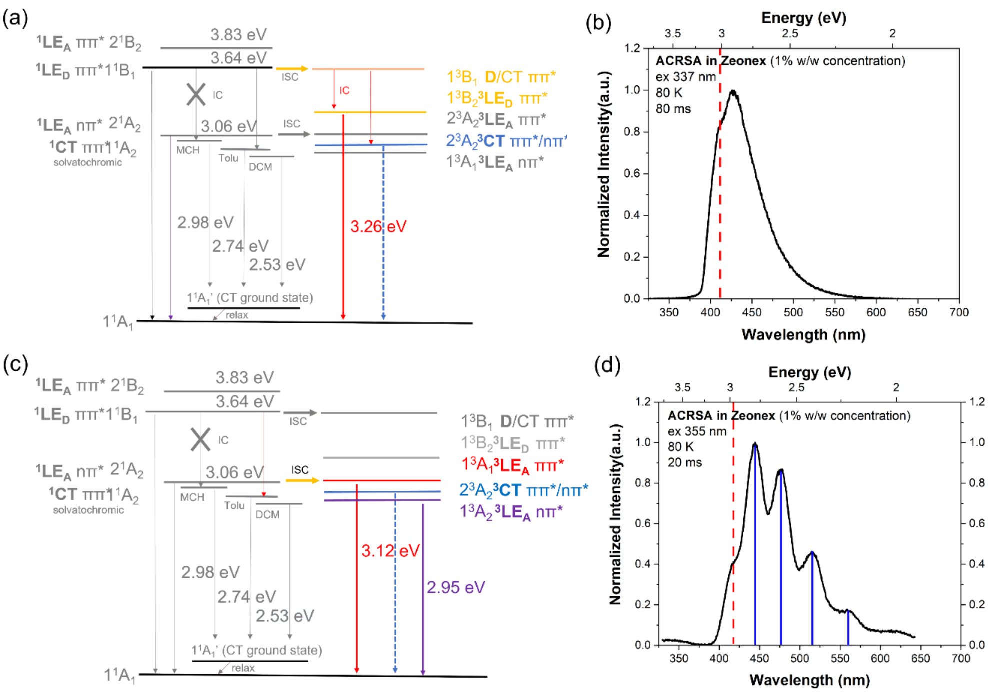

Figure S9: (a) Energy diagram representing the pathway for the decay of triplet excited states of **ACRSA** when excited at 337 nm (into the 1^1B_1 transition). (b) Phosphorescence spectra of **ACRSA** in zeonex at 1% concentration, exciting at 337 nm. (c) Energy diagram representing the pathway for the decay of triplet excited states of **ACRSA** when excited at 355 nm (the direct mixed 2^1A_2 and 1^1A_2 transitions). (b) Phosphorescence spectra of **ACRSA** in zeonex at 1% concentration, exciting at 355 nm. The nomenclature used here was adopted from Lyskov and Marian.[1]

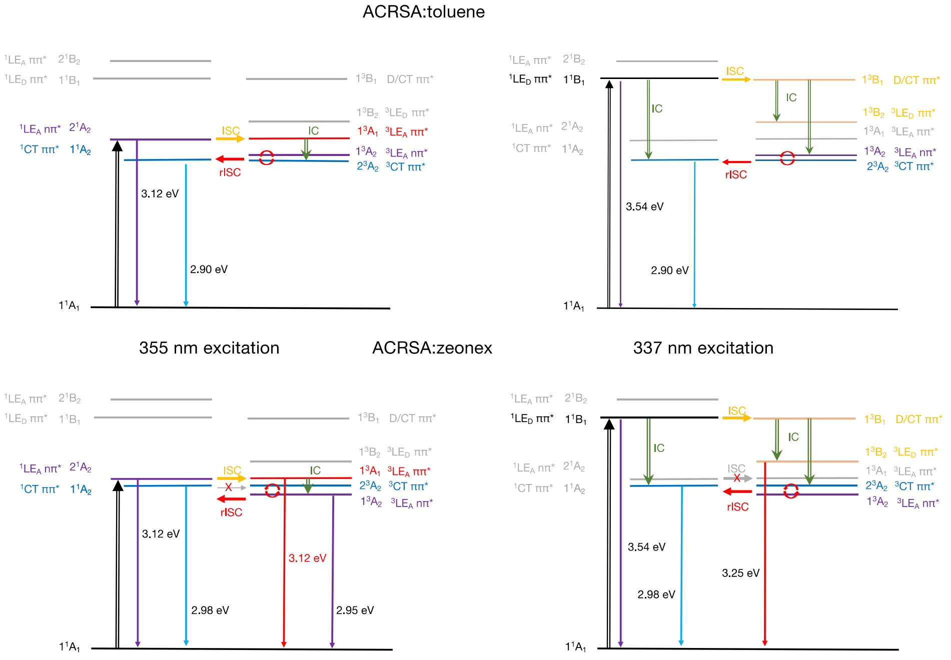

Figure S10: Proposed experimental energy level pathways for **ACRSA** in toluene and zeonex matrix (at 1% concentration) when excited at 337 nm and at 355 nm. The nomenclature used here was adopted from Lyskov and Marian.[1]

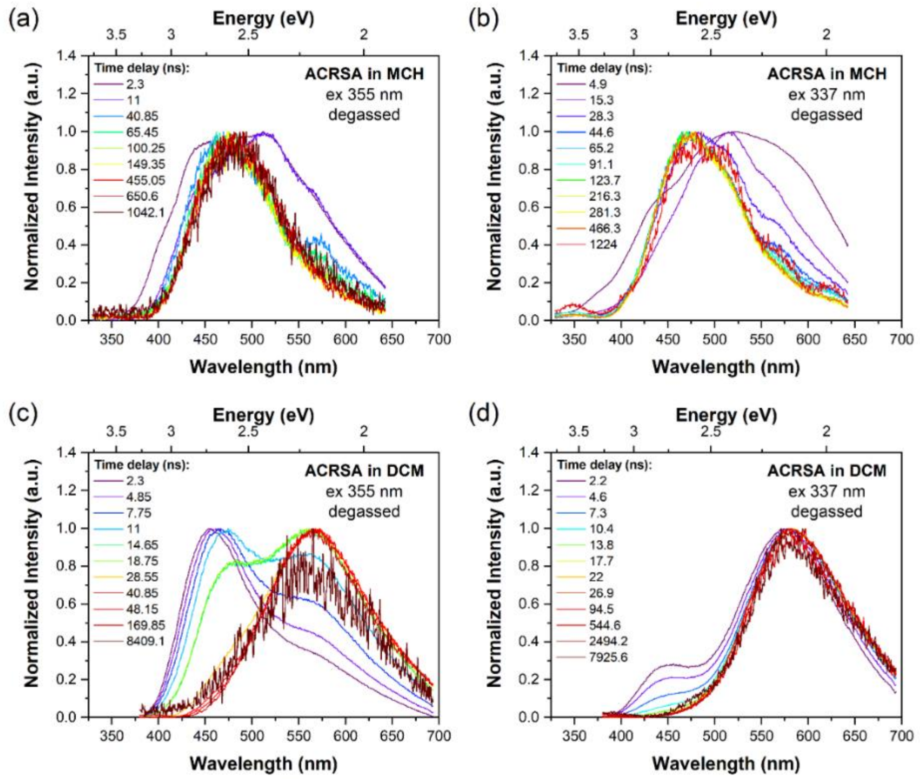

Figure S11: Normalized time resolved photoluminescence spectra of **ACRSA** in MCH and DCM, excited at: (a-c) 355 nm (the direct mixed 2^1A_2 and 1^1A_2 transitions) and (b-d) 337 nm (into the 1^1B_1 transition). All measurements were performed in degassed solutions (with a concentration of 50 μM) at room temperature.

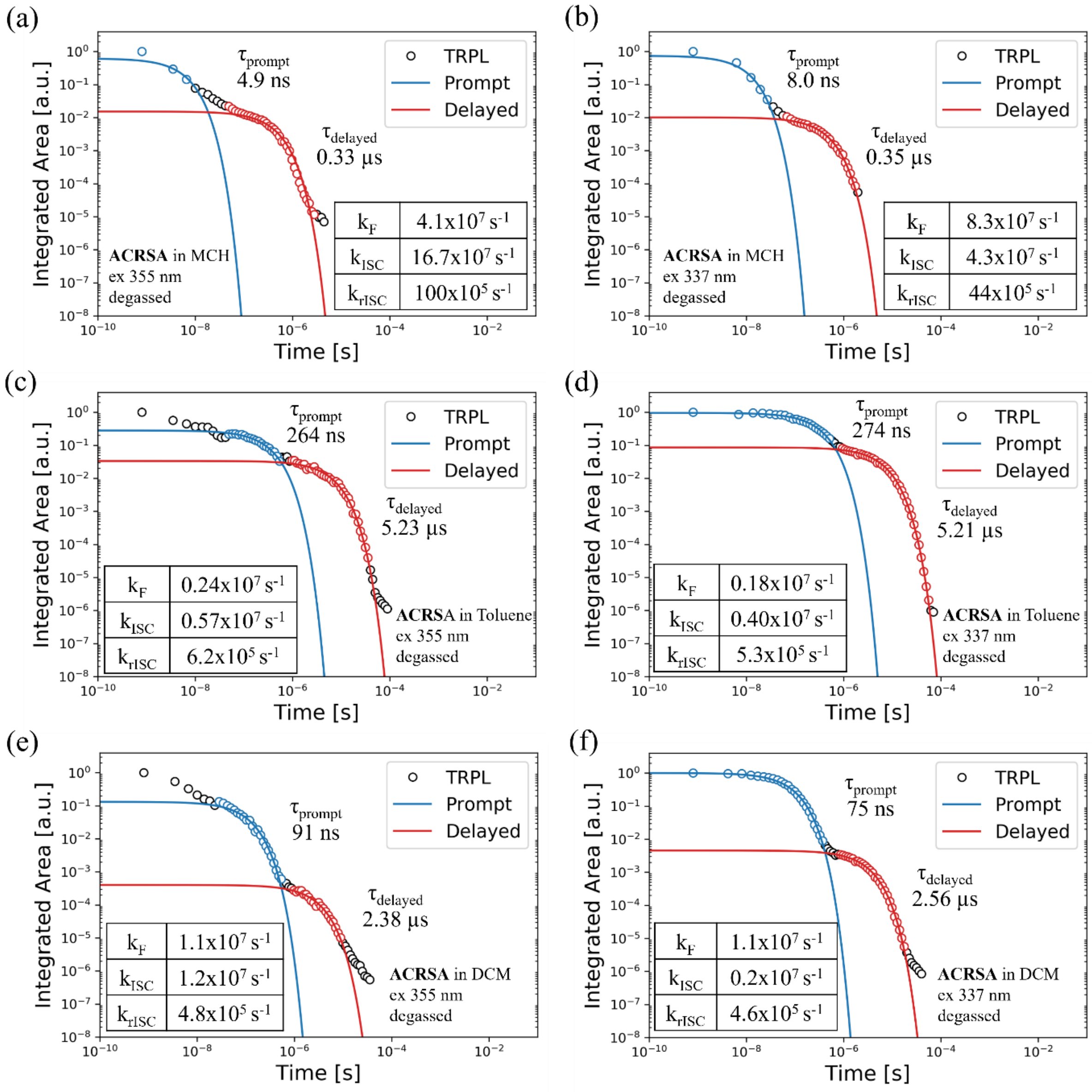

Figure S12: Time resolved photoluminescence decays of **ACRSA** in degassed MCH, toluene and DCM solutions (concentration 50 μM) excited at 337 nm into the 1^1B_1 transition (a, c, e) and at 355 nm into the direct mixed 2^1A_2 and 1^1A_2 transitions (b, d, f). The data is fitted using a kinetic model described by Haase *et al*[2].

Scheme S1: Mechanism describing the ISC and rISC pathways from different initial singlet excited state.

ISC and rISC via the 1^1B_1 donor state (337 nm) excitation:

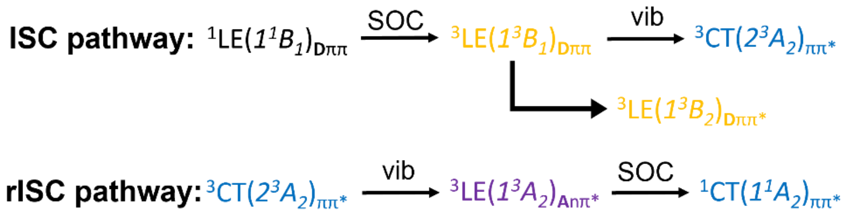

ISC and rISC via the 2^1A_2 acceptor state (355 nm) excitation:

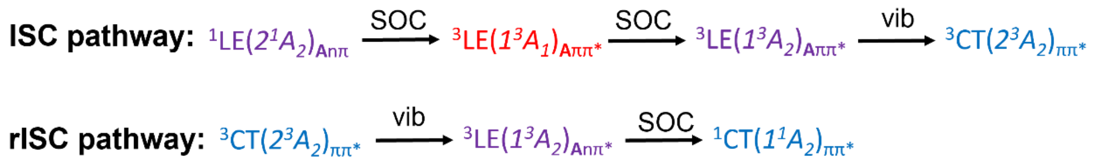

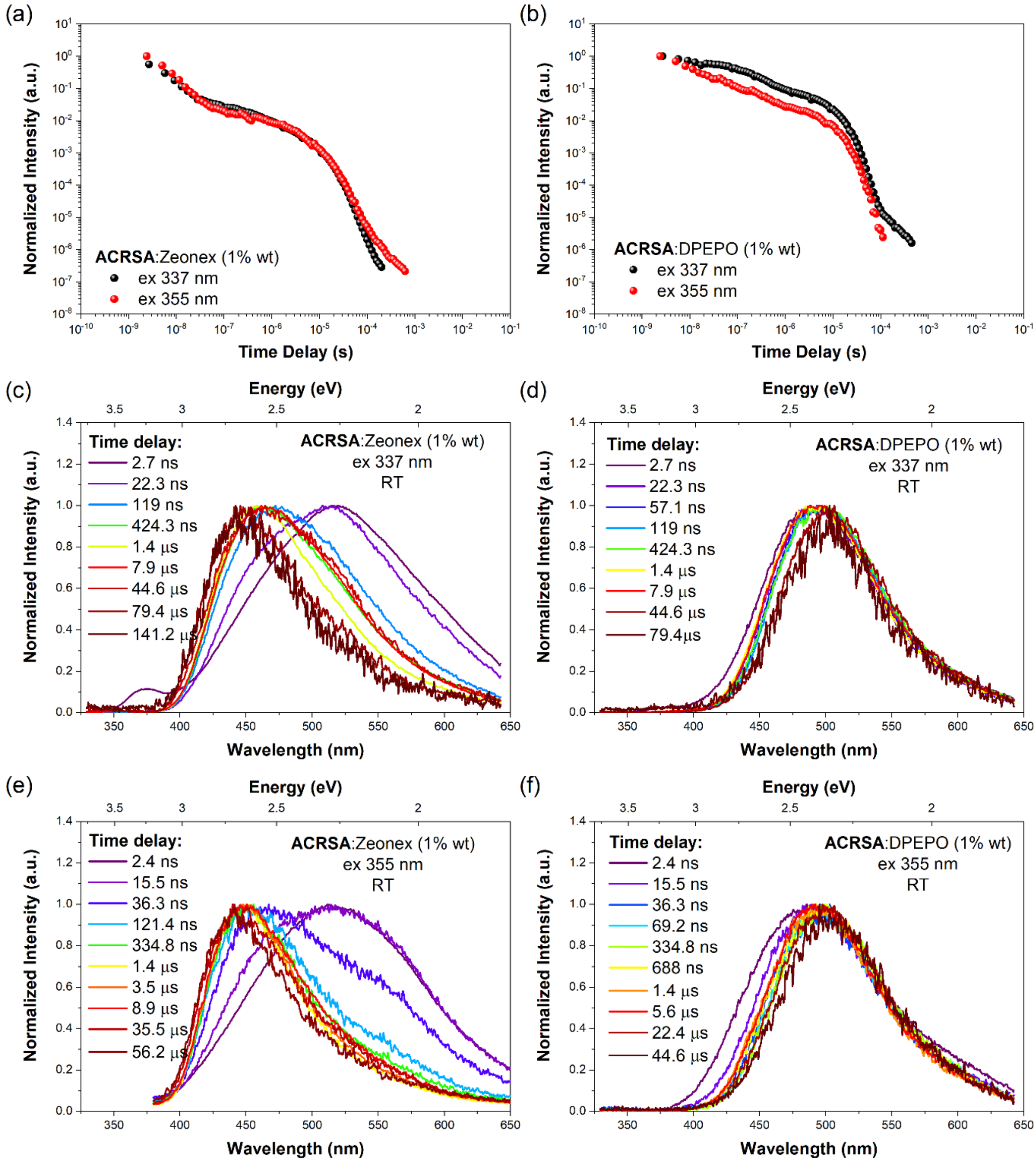

Figure 13: Time resolved photoluminescence decays (a-b) and normalized time resolved photoluminescence spectra of **ACRSA** in Zeonex and DPEPO, excited at: (c-d) 337 nm (into the 1^1B_1 transition) and (e-f) 355 nm (the direct mixed 2^1A_2 and 1^1A_2 transitions). All measurements were performed at room temperature.

Table S1: Lifetimes and amplitudes as a function of temperature. All data obtained by fitting photoluminescence decay of **ACRSA** in Zeonex (1%) and DPEPO (1%); with 330 nm excitation wavelength

**Temperatures (K)**	**ACRSA:**Zeonex **1%**		**ACRSA:**DPEPO **1%**
	**τ_1 (μs) / A_1 (%)**	**τ_2 (μs) / A_2 (%)**	**τ_1 (μs)**
290	4.6/81	13.7/19	9.5
275	4.8/81	16.6/19	10.3
250	7.9/81	22.3/19	11.7
225	11.7/92	39.4/8	13.1
200	15.4/92	52.9/8	15.3
175	20.3/93	85.8/7	18.6
150	27.1/92	122.9/8	22.8
125	37.9/88	161.7/12	28.6
100	47.4/64	163.5/36	53.4

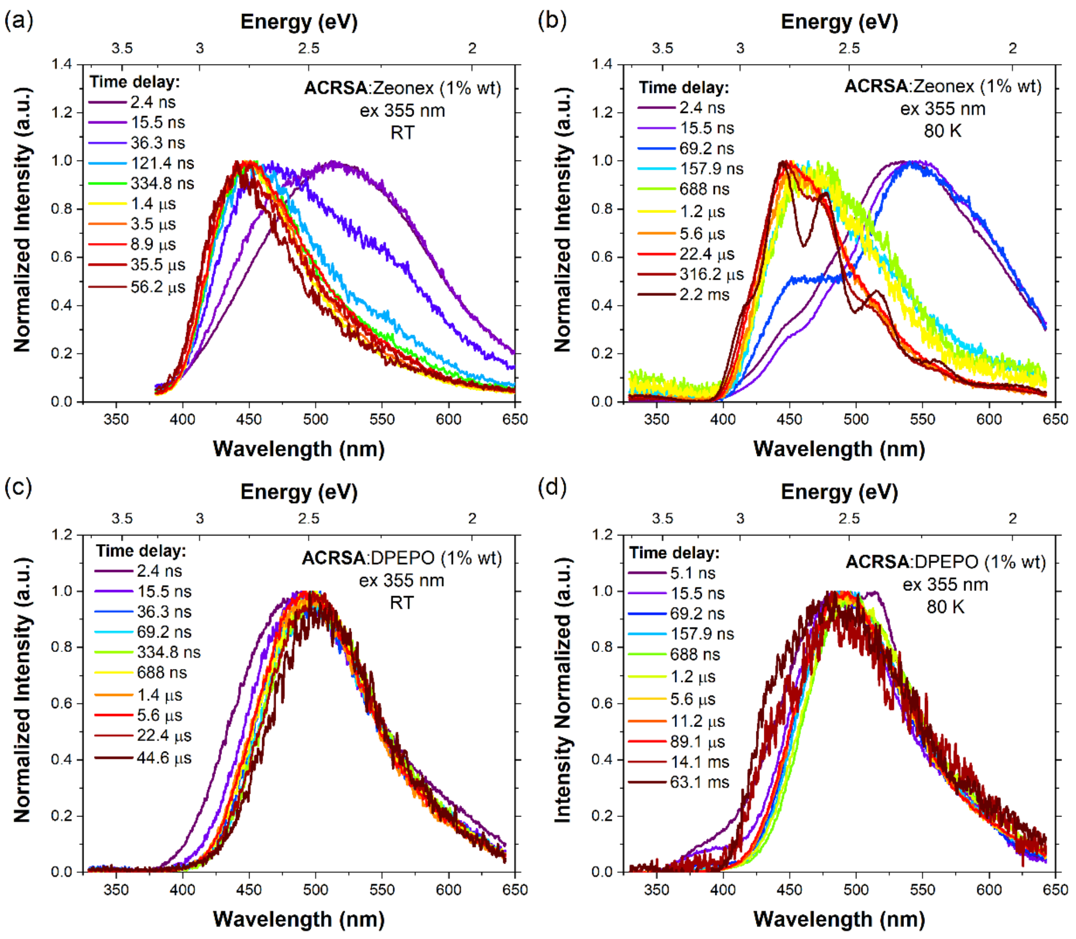

Figure S14: Normalized time resolved photoluminescence spectra of **ACRSA** in (a-b) zeonex and (c-d) DPEPO host matrices at 1% concentration, measured at room temperature (RT) and 80 K respectively. All measurements were performed using an excitation source of 355 nm.

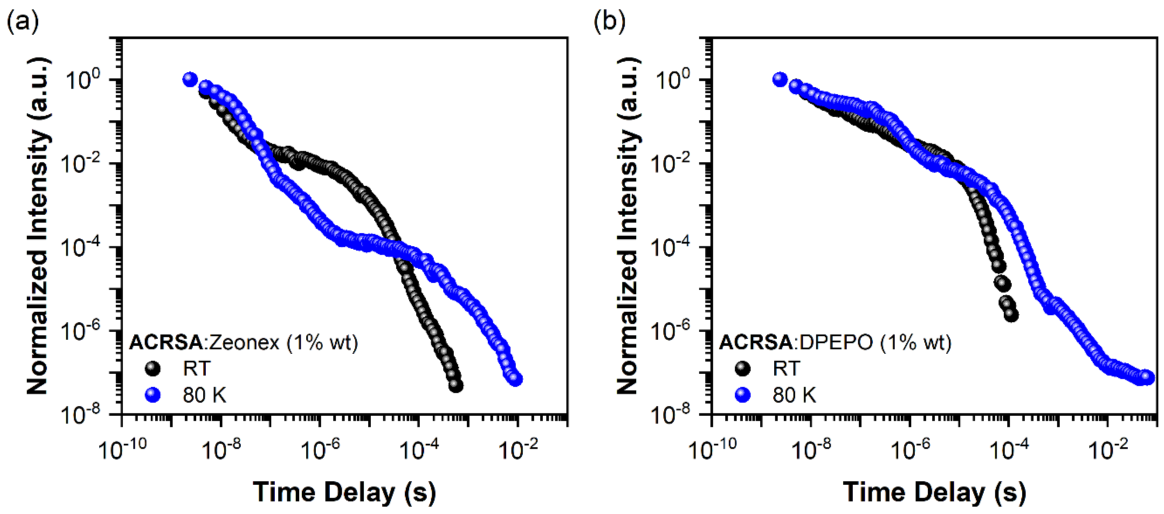

Figure S15: Time resolved photoluminescence decays of **ACRSA** in (a) zeonex and (b) DPEPO host matrices at 1% concentration, measured at room temperature (RT) and 80 K. All measurements were performed using an excitation source of 355 nm.

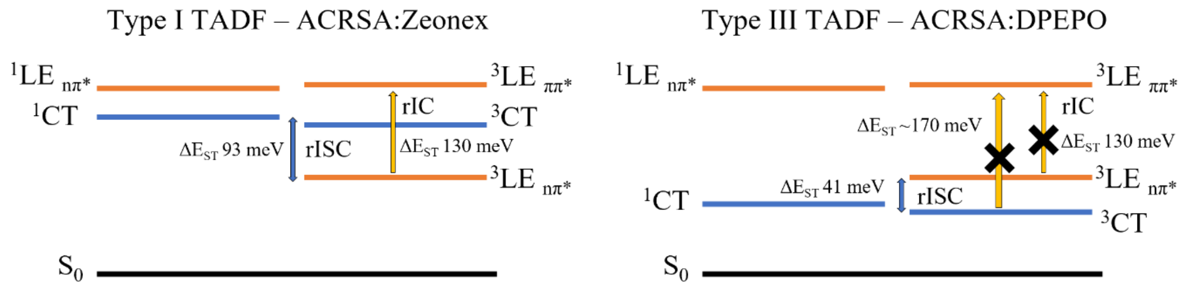

Figure S16: Schematic energy level diagrams of **ACRSA** in Zeonex and DPEPO, based on the optical singlet-triplet gap.

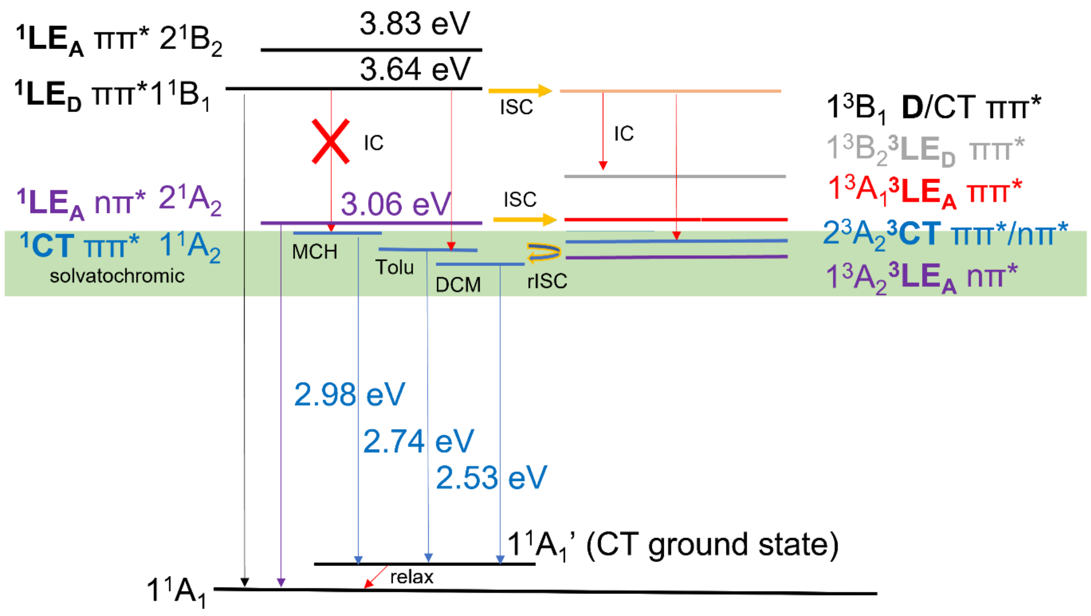

Figure S17: Energy level scheme for ACRSA in different solvents. Following the nomenclature used by Lyskov and Marian[1]. The energy range for solvatochromic states is highlighted in green.

Additional photophysical properties of the donor (acridine) and acceptor (anthrone) units to compare with the ACRSA molecule:

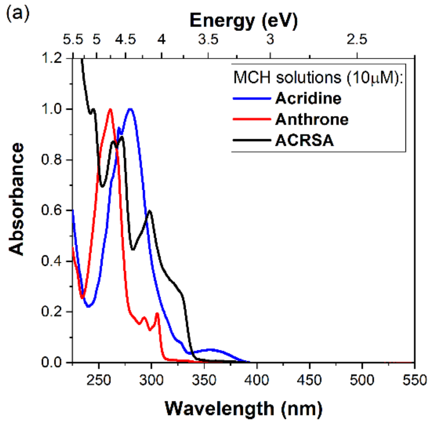

Figure S18: Absorption spectra of the donor (acridine), acceptor (anthrone) units and the **ACRSA** molecule in (a) MCH solutions at 10 μM and (b) zeonex matrix (1% wt).

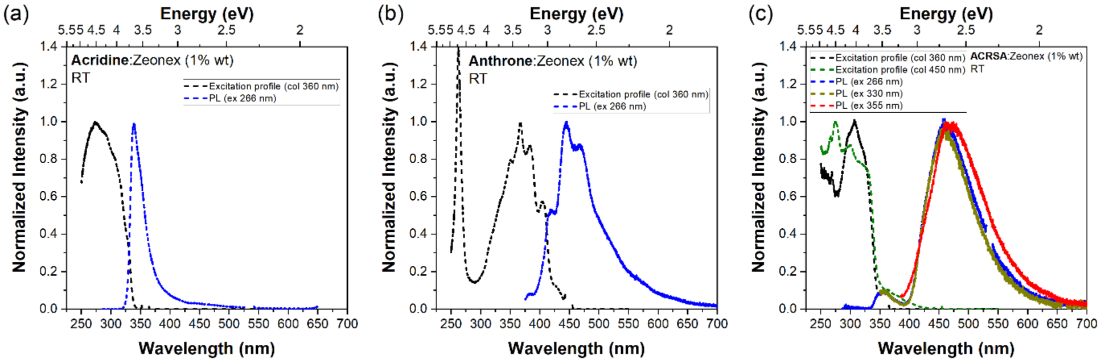

Figure S19: Normalized excitation profile and photoluminescence spectra of the donor (acridine), acceptor (anthrone) units and the **ACRSA** molecule in zeonex matrix (1% wt). Measurement was performed at room temperature.

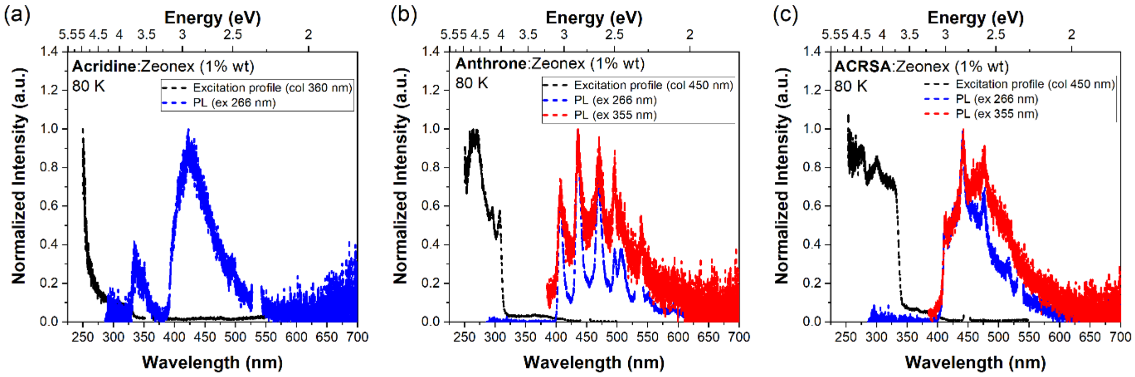

Figure S20: Normalized excitation profile and photoluminescence spectra of the donor (acridine), acceptor (anthrone) units and the **ACRSA** molecule in zeonex matrix (1% wt). Measurement was performed at 80 K.

References:

(1) Lyskov, I.; Marian, C. M. Climbing up the Ladder: Intermediate Triplet States Promote the Reverse Intersystem Crossing in the Efficient TADF Emitter ACRSA. *J. Phys. Chem. C* **2017**, *121* (39), 21145–21153.

(2) Haase, N.; Danos, A.; Pflumm, C.; Morherr, A.; Stachelek, P.; Mekic, A.; Brütting, W.; Monkman, A. P. Kinetic Modeling of Transient Photoluminescence from Thermally Activated Delayed Fluorescence. *J. Phys. Chem. C* **2018**, *122* (51), 29173–29179.
